# A multilocus approach for accurate variant calling in low-copy repeats using whole-genome sequencing

**DOI:** 10.1093/bioinformatics/btad268

**Published:** 2023-06-30

**Authors:** Timofey Prodanov, Vikas Bansal

**Affiliations:** Bioinformatics and Systems Biology Graduate Program, University of California San Diego, La Jolla, CA 92093, United States; Institute for Medical Biometry and Bioinformatics, Medical Faculty, Heinrich Heine University, Düsseldorf 40225, Germany; Center for Digital Medicine, Heinrich Heine University, Düsseldorf 40225, Germany; School of Medicine, University of California San Diego, La Jolla, CA 92093, United States

## Abstract

**Motivation:**

Low-copy repeats (LCRs) or segmental duplications are long segments of duplicated DNA that cover > 5% of the human genome. Existing tools for variant calling using short reads exhibit low accuracy in LCRs due to ambiguity in read mapping and extensive copy number variation. Variants in more than 150 genes overlapping LCRs are associated with risk for human diseases.

**Methods:**

We describe a short-read variant calling method, ParascopyVC, that performs variant calling jointly across all repeat copies and utilizes reads independent of mapping quality in LCRs. To identify candidate variants, ParascopyVC aggregates reads mapped to different repeat copies and performs polyploid variant calling. Subsequently, paralogous sequence variants that can differentiate repeat copies are identified using population data and used for estimating the genotype of variants for each repeat copy.

**Results:**

On simulated whole-genome sequence data, ParascopyVC achieved higher precision (0.997) and recall (0.807) than three state-of-the-art variant callers (best precision = 0.956 for DeepVariant and best recall = 0.738 for GATK) in 167 LCR regions. Benchmarking of ParascopyVC using the genome-in-a-bottle high-confidence variant calls for HG002 genome showed that it achieved a very high precision of 0.991 and a high recall of 0.909 across LCR regions, significantly better than FreeBayes (precision = 0.954 and recall = 0.822), GATK (precision = 0.888 and recall = 0.873) and DeepVariant (precision = 0.983 and recall = 0.861). ParascopyVC demonstrated a consistently higher accuracy (mean *F*_1_ = 0.947) than other callers (best *F*_1_ = 0.908) across seven human genomes.

**Availability and implementation:**

ParascopyVC is implemented in Python and is freely available at https://github.com/tprodanov/ParascopyVC.

## 1 Introduction

Advances in DNA sequencing technologies have transformed the ability to sequence genomes, particularly human genomes. Accurate variant calling is of crucial importance for virtually all applications of high-throughput DNA sequencing including disease genetics and cancer. Small variants such as single nucleotide variants (SNVs) and short indels represent the most abundant type of variants in human genomes and a number of methods (e.g. Samtools, FreeBayes, GATK) have been developed to call such variants from DNA sequence data (DePristo et al. 2011; Li 2011; Garrison and Marth 2012; Kim et al. 2018; Poplin et al. 2018a). Most of these methods leverage statistical techniques to discriminate true genetic variants from artifacts due to errors in reads and exhibit high accuracy in unique regions of the human genome that are callable using short reads. The Genome-in-a-Bottle Consortium (GIAB) has developed benchmark sets for small variants across seven genomes that are useful for benchmarking and optimizing variant calling methods (Zook et al. 2014, 2016, 2019).

However, a significant portion of the human genome is repetitive (Treangen and Salzberg, 2011) and remains challenging for variant calling using short read sequencing. In particular, segmental duplications—also known as low-copy repeats—that have been estimated to cover ∼5% of the human genome (Bailey et al. 2002), are problematic for variant calling due to ambiguity in read mapping (Koboldt 2020). Recent analysis of segmental duplications in a complete human genome (T2T-CHM13) has revealed that such repeats cover an estimated 7% of the genome (Vollger et al. 2022). Short reads derived from such regions align to multiple locations and are assigned low mapping quality scores by mapping tools (Li and Durbin 2009; Li 2018). Such reads are typically discarded during variant calling to avoid false-positive variant calls (DePristo et al. 2011; Garrison and Marth 2012) resulting in low sensitivity. Until recently, the GIAB benchmark calls excluded most of these low-copy repeat (LCR) regions due to the difficulty in accurate variant calling. As a result, the SNV concordance of two state-of-the-art variant callers was observed to be 99.7% within GIAB high-confidence regions compared to 76.5% outside (Krusche et al. 2019).

LCRs overlap hundreds of protein-coding genes (Bailey et al. 2002) in the human genome. A recent analysis of sequence homology for coding regions in the human genome identified 7691 exons in 1168 genes, which have partial or complete sequence homology (>98%) to one or more loci (Mandelker et al. 2016). Copy number and sequence variants in 193 of these 1168 genes are associated with rare Mendelian disorders, inherited cancers and complex diseases. Therefore, improving the accuracy of variant calling in LCRs has great clinical importance. One well-studied example of a disease-associated gene that overlaps a LCR is the *SMN1* gene—mutations in this gene cause spinal muscular atrophy, a severe childhood disease. *SMN1* is entirely duplicated and has close to 99.9% sequence similarity with its homologous gene *SMN2* (Lefebvre et al. 1995). Furthermore, *SMN1/2* is prone to frequent copy number changes which further complicates the task of small variant calling (Chen et al. 2020; Lopez-Lopez et al. 2020).

Sequence differences between the different repeat copies of an LCR are commonly referred to as paralogous sequence variants (PSVs). PSVs represent the only source of information to differentiate between repeat copies of a LCR and read mappers implicitly use PSVs to map reads in LCRs. However, not all PSVs represent fixed differences between repeat copies and many correspond to common variants (Sudmant et al. 2010; Mueller et al. 2013). For example, the reference human genome sequence for the *SMN1* gene differs from *SMN2* gene at 24 positions across 28 kb of DNA sequence (sequence similarity >0.999), but only 8 of these 24 positions correspond to fixed differences in human populations (Chen et al. 2020). A similar phenomenon has been observed for another disease-associated gene, *PMS2*, that frequently exchanges DNA sequence with its nearby pseudogene, *PMS2CL*, making it difficult to distinguish between the two repeat copies (Clendenning et al. 2006; Gould et al. 2018). Using polymorphic PSVs for mapping reads in LCRs can result in incorrect read mapping and reduce the accuracy of variant calling. We have recently developed a probabilistic method for estimating the paralog-specific copy number of genes at LCRs that also estimates the reference allele frequencies at PSVs (Prodanov and Bansal 2022) using population data. Our analysis of 2500 genomes from the 1000 Genomes Project (1000 Genomes Project Consortium et al. 2015) showed that the frequency of PSVs that represent fixed or almost fixed differences between repeat copies varies widely across LCR loci and also across populations at the same locus (Prodanov and Bansal 2022).

All state-of-the-art variant calling methods are designed to analyze genomic regions individually and discard or downweight reads with low mapping quality. In LCRs, a significant fraction of reads can be mapped to the incorrect location or have zero mapping quality. Therefore, variant calling on each repeat copy individually cannot achieve high precision and recall. For high accuracy, joint analysis of reads mapped to all homologous repeat copies is necessary. This strategy has proven to be successful in estimating copy number with high accuracy at LCR loci (Sudmant et al. 2010; Prodanov and Bansal 2022). Several approaches that re-map reads to a single copy of the repeat (and mask out other copies before read mapping) and perform variant calling with higher ploidy (Cummings et al. 2017; Gould et al. 2018; Boisson et al. 2019; Ebbert et al. 2019) have been developed to enable the detection of disease mutations in duplicated genes. Although this approach increases sensitivity for variant detection, it is challenging to estimate the genotypes for each variant across the different repeat copies.

In this article, we describe a multilocus variant calling method, ParascopyVC, that addresses three challenges associated with variant calling in LCRs: (i) ambiguity in read mapping, (ii) presence of PSVs that are polymorphic, and (iii) presence of copy number changes. ParascopyVC combines two methodological innovations: (i) aggregating reads mapped to different repeat copies to enable highly sensitive detection of variant sites and (ii) identification of informative PSVs that can differentiate repeat copies for paralog-specific genotyping at variant sites. For variant discovery, it leverages an existing variant calling tool, FreeBayes, to jointly analyze all reads mapped to the different repeat copies. ParascopyVC uses paralog-specific copy number estimated from WGS reads using Parascopy (Prodanov and Bansal 2022) to model the ploidy of paralog-specific genotypes. We benchmarked ParascopyVC using simulated data and whole-genome WGS data for seven human individuals, for which high-quality variant call sets in LCRs were recently published by the GIAB consortium. Our results demonstrate that ParascopyVC significantly outperforms state-of-the-art variant callers both in precision and recall in LCR regions.

## 2 Materials and methods

We consider a LCR region *L* with a total of *n* repeat copies (including the region *L*) in the reference genome. The input data are a set of WGS reads mapped to the reference genome (assumed to be diploid for our method). Let *R* denote the subset of reads that overlap any of the repeat copies of *L*. The goal of small variant calling is to identify positions in the regions of interest (the repeat copies) that differ from the reference and to estimate the most probable genotype for each variant. Standard variant calling tools analyze each repeat copy separately and identify variants by examining reads aligned to the reference genome. However, in LCRs, many reads are mapped ambiguously (multiple identical alignment possibilities) or even mapped to an incorrect repeat copy. This results in a large number of both false negative and false positive variant calls. To solve this problem, ParascopyVC jointly analyzes reads mapped to all repeat copies for variant discovery and genotyping.

### 2.1 Definitions

We introduce a set of terms and notations that will be useful for the description of ParascopyVC. Since we consider variants jointly across all repeat copies, a variant is characterized by an *n*-tuple of genomic positions (one for each repeat copy) and an allele set *A*. These “paralogous positions” can be identified using multiple sequence alignment of the reference sequence for the repeat copies (Prodanov and Bansal 2022). In the reference genome, variant *v* exhibits allele $a_{vi}^{*}$ on *i*th repeat copy of the duplication. If the variant *v* is a PSV, then $a_{vi}^{*}\neq a_{vj}^{*}$ for some *i*, *j*. For a PSV *v*, $f_{vi}\in (0,1)$ is the frequency of the reference allele $a_{vi}^{*}$ in the population on the *i*th repeat copy.

Paralog-specific copy number of a sample *s* is a tuple ${\left(c_{si}\right)}_{i=1}^{n},\;c_{si}\in N_{\geq 0}$. Each element of the tuple $c_{si}$ stands for the number of times the *i*th repeat copy appears in the genomic sequence of the sample. Aggregate copy number ${\hat{c}}_{s}=\sum_{i=1}^{n}c_{si}$ is the sum of paralog-specific copy numbers across all repeat copies. In certain cases, paralog-specific copy number values are not available for all repeat copies. In such cases, we call variants on *extended* repeat copies (see Supplementary Methods 2.1 for details).

Aggregate genotype ${\hat{g}}_{vs}$ of a variant *v* in the sample *s* is a multiset of ${\hat{c}}_{s}$ alleles—such genotype collects variant alleles across all repeat copies without any specific order and can contain the same allele many times. A paralog-specific genotype $g_{vs}$ is a tuple of *n* allele multisets, where the *i*th multiset contains $c_{si}$ alleles. Paralog-specific genotype of a variant refers to the *n*-tuple of genotypes for the variant across the *n* repeat copies. We will use multiplicity function μ(z,Z) to denote the number of occurrences of an element *z* in the multiset *Z*.

As an example, consider a two-copy LCR and a sample *s* with aggregate copy number ${\hat{c}}_{s}=5$ and paralog-specific copy number $c_{s}=(3,2)$. One possible aggregate genotype of a variant *v* with two alleles $A_{v}=\{G,T\}$ would be ${\hat{g}}_{vs}=G/G/G/T/T$ and one possible paralog-specific genotype would be $g_{vs}=(G/T/T,\;G/G)$. Each paralog-specific genotype is associated with a single aggregate genotype, obtained by combining all multisets $g_{\mathrm{vsi}}$. A paralog-specific genotype $g_{vs}$ is *reference-compatible*, if, for all *i*, multiset $g_{\mathrm{vsi}}$ contains only the reference allele $a_{vi}^{*}$; in other words, if $\mu (a_{vi}^{*},g_{\mathrm{vsi}})=c_{si}$.

### 2.2 Algorithm

We assume that PSVs have been identified in advance using multiple sequence alignment of the reference sequences for the repeat copies. We also assume that paralog-specific and aggregate copy numbers for the repeat copies are known for the sample. Our previously developed copy number estimation tool, Parascopy (Prodanov and Bansal 2022), identifies PSVs; estimates allele frequencies *f* for each PSV; and calculates paralog-specific copy number using WGS data. For a region *L* with *n* copies, variant calling using ParascopyVC proceeds in three steps.

**Figure 1.**
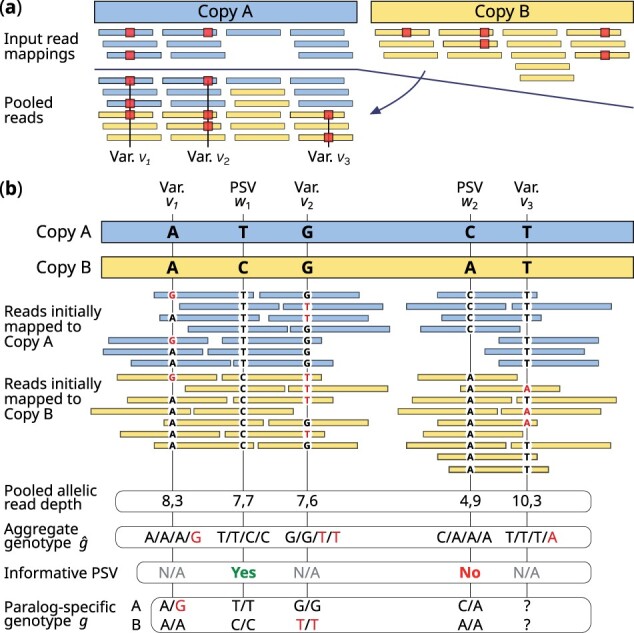
Illustration of multilocus variant calling approach in LCRs. The figure shows a two-copy LCR with repeat copies A (blue) and B (yellow). (a) The reads initially aligned to Copy B are remapped to Copy A for aggregate variant calling and three variants ($v_{1},v_{2}$, and $v_{3}$) are identified. (b) Reference sequences of copies A and B differ at two PSVs ($w_{1}$ and $w_{2}$). Using population reference allele frequencies and aggregate genotype information, the PSV $w_{1}$ is determined to be informative while $w_{2}$ is not. Reads that overlap variant $v_{1}$ and the informative PSV $w_{1}$ indicate that the *G* allele of variant $v_{1}$ is present on copy *A* (paralog-specific genotype for $v_{1}=(A/G,A/A)$). Paralog-specific genotype for variant $v_{3}$ is not inferred due to the lack of an informative PSV.

The first step is the identification of candidate variant sites with high sensitivity using “aggregate variant calling”. For this, we re-map reads from all repeat copies to a single copy (*L*) and use polyploid variant calling—with ploidy equal to the aggregate copy number—to call variants. Note that this re-mapping is done locally and does not involve global re-mapping of reads. This process is illustrated in Fig. 1a for a two-copy duplication. This enables the use of all reads, regardless of mapping quality, for sensitive variant detection.

Second, we “identify informative PSVs”—PSVs whose paralog-specific genotypes are identical to the reference for the sample. For each variant v∈V, we estimate the most likely aggregate genotype ${\hat{g}}_{vs}$ using the aggregated reads independent of other variants. For PSVs, we calculate paralog-specific genotype probabilities based on the aggregate genotype likelihoods and by using reference allele frequencies $f_{v}$ in order to calculate paralog-specific genotype priors. PSVs with reference-compatible paralog genotypes are marked as *informative*. In the example shown in Fig. 1b, PSV $w_{1}$ is informative (paralog-specific genotype = (T/T,C/C), same as reference), while PSV $w_{2}$ is not.

The third and final step in ParascopyVC is “paralog-specific genotyping” of all variants using informative PSVs and paired reads that overlap both informative PSVs and variants. For variant $v_{1}$ in Fig. 1b, the most-likely aggregate genotype is A/A/A/G. Hence, there are two possible paralog-specific genotypes: (A/A, A/G) and (A/G, A/A) for this variant. Since there are reads that overlap both PSV $w_{1}$ and variant $v_{1}$ have the alleles *G* and *T* at the two sites respectively, we can estimate the paralog-specific genotypes for $v_{1}$ to be (A/G,A/A). Note that paralog-specific genotypes cannot be estimated for variants that are not covered by reads that also overlap informative PSVs, e.g. variant $v_{3}$ in Fig. 1b.

Next, we describe the mathematical details of the three steps of the algorithm.

### 2.3 Step 1: aggregate variant calling

During its first stage, the copy number estimation method Parascopy (Prodanov and Bansal 2022) identifies regions homologous to *L* using a pre-computed homology table and re-maps reads from them back to *L*. Our variant calling method, ParascopyVC, uses this same approach to re-map reads and then runs an existing variant calling tool FreeBayes (Garrison and Marth 2012) on the pooled reads with ploidy ${\hat{c}}_{s}$. We modify the resulting set of aggregate variants by removing all variants with low quality (<1), and by extending the set to include all PSVs. For each variant *v*, we calculate the allelic read depth $X_{sv}$ across the variant alleles $A_{v}$ in the pooled reads $R_{sv}$ overlapping the variant. The aggregate genotype probabilities are calculated using $X_{sv}$ and the multinomial distribution (MN) as follows: and where ϵ is the error rate, $A_{v}$ is the full set of alleles, $\left|\bar{r}\right|$ is mean read length, |a| is the allele length, and μ is the multiplicity function. The calculations are consistent with the polyploid genotype likelihood calculations used in FreeBayes (Garrison and Marth 2012). Finally, we use Bayes’ theorem to get the aggregate genotype probability:


(1) $$P(X_{vs}\;|\;{\hat{g}}_{vs})=P_{\text{MN}}(X_{vs};\;p(a_{1},\;{\hat{g}}_{vs}),\ldots ,p(a_{\left|A_{v}\right|},\;{\hat{g}}_{vs})),$$

(2) $$\text{where}\;p(a,\;\hat{g})=\frac{(|\bar{r}|-|a|-1)\cdot \max\left\{\epsilon \cdot {\hat{c}}_{s},\;\mu (a,\hat{g})\right\}}{\sum_{a^{\prime }\in A_{v}}\left(|\bar{r}|-|a^{\prime }|-1\right)\cdot \max\left\{\epsilon \cdot {\hat{c}}_{s},\;\mu (a^{\prime },\hat{g})\right\}}$$

(3) $$P({\hat{g}}_{vs}\;|\;X_{vs})=\frac{p({\hat{g}}_{vs})\cdot P(X_{vs}\;|\;{\hat{g}}_{vs})}{\sum_{{\hat{g}}^{\prime }\in {\hat{G}}_{vs}}p({\hat{g}}^{\prime })\cdot P(X_{vs}\;|\;{\hat{g}}^{\prime })}.$$

Note that the set of aggregate genotypes ${\hat{G}}_{vs}$ depends on the set of alleles $A_{v}$ and aggregate copy number ${\hat{c}}_{s}$. Additionally, we select equal aggregate genotype priors $p(\hat{g})=1/|{\hat{G}}_{vs}|$. In order to improve genotyping accuracy, allele observations with base qualities less than 10 and all partial allele observations are discarded.

### 2.4 Step 2: identify informative PSVs

For PSVs, the prior probability of paralog-specific genotypes can be obtained using the PSV reference allele frequencies *f*:


(4) $$P(g_{vs};\;f_{v})=\prod_{i=1}^{n}f_{vi}^{\;\mu \left(a_{vi}^{*},\;g_{\mathrm{vsi}}\right)}\times {\left(1-f_{vi}\right)}^{\;c_{si}-\mu \left(a_{vi}^{*},\;g_{\mathrm{vsi}}\right)}.$$

In other words, the prior of the paralog-specific genotype *g* is a product of either $f_{vi}$ or $\left(1-f_{vi}\right)$ depending on the match between the genotype alleles and the reference alleles of the PSV *v*.

For each PSV, we calculate the probability of paralog-specific genotype $g_{vs}$ based on the PSV allele frequencies $f_{v}$ and the probability of its associate aggregate genotype ${\hat{g}}_{vs}$: where $G(\hat{g})$ is the full set of paralog-specific genotypes associated with the aggregate genotype $\hat{g}$.


(5) $$\begin{array}{c} P(g_{vs}\;|\;X_{vs};\;f_{v})=P({\hat{g}}_{vs}\;|\;X_{vs})\cdot P(g_{vs}\;|\;{\hat{g}}_{vs})\\ =P({\hat{g}}_{vs}\;|\;X_{vs})\cdot \frac{P(g_{vs};\;f_{v})}{\sum_{g^{\prime }\in G({\hat{g}}_{vs})}P(g^{\prime };\;f_{v})}. \end{array}$$

We say that the PSV *v* is *informative* for a sample *s* if the most likely paralog-specific genotype for sample *s* has high posterior probability (≥0.99 by default) and is reference-compatible. Such PSVs can be used to differentiate reads originating from different repeat copies with high confidence.

Filtering out noninformative PSVs:

The estimation of paralog-specific genotypes for PSVs does not utilize information from read-pairs that cover more than one PSV since this greatly increases the computational complexity of the likelihood calculations. As a result, read-pairs that cover multiple informative PSVs can sometimes conflict with the reference genotype for a pair of informative PSVs. Such conflicts indicate that one of the two PSVs is not informative. For each pair of informative PSVs covered by at least three reads, we tabulate the read-allele counts for the pair and use a one-tailed Binomial test ($\pi =2\epsilon -\epsilon^{2}$ and *P* value threshold of $10^{-3}$) to identify conflicting PSVs.

Next, we construct an undirected graph on the set of informative PSVs with edges between conflicting PSVs. Then we aim to keep a subset of informative PSVs such that the remaining graph contains no edges and $\sum {\tilde{f}}_{v}$ is maximal, where ${\tilde{f}}_{v}=\min_{i=1}^{n}f_{vi}$—the minimal frequency *f* of the PSV *v* across all repeat copies. This problem is equivalent to an NP-complete weighted maximum clique problem (Karp 1972). Therefore, we employ the following greedy heuristic: until the graph is edgeless, we iteratively remove the PSV *v* that has the maximal number of edges multiplied by ${\left(1-{\tilde{f}}_{v}\right)}^{1/2}$.

### 2.5 Step 3: estimate paralog-specific genotypes

For new variants, we infer paralog-specific genotypes using read-pairs that overlap the variant and an informative PSV (or, possibly, a nonduplicated region of the genome). For each read-pair *r*, we determine the set of possible read locations (see Supplementary Methods 2.2) across all repeat copies, and then estimate read-pair location probabilities $p_{r}(i)$ using the informative PSVs (denoted by $W_{r}$) covered by the read pair *r*. Here, $p_{r}(i)$ is the probability that location *i* is the true origin of read-pair *r*. Suppose a PSV *v* has allele $a_{vi}$ on the *i*th repeat copy, then we assign $P(a_{vi}\;|\;r)$ to either 1−ϵ or ϵ (error rate) depending upon whether the read sequence matches or does not match the allele $a_{vi}$, respectively. Location priors are estimated based on the paralog-specific copy numbers $c_{si}$, which results in the following formula for $p_{r}(i)$:


(6) $$p_{r}(i)=\frac{c_{si}\prod_{v\in W_{r}}P(a_{vi}\;|\;r)}{\sum_{j=1}^{n}c_{sj}\prod_{v\in W_{r}}P(a_{vj}\;|\;r)}.$$

If a PSV $v\in W_{r}$ is missing from one of the repeat copies *j* because the copy is shorter than others, we penalize location probability $p_{r}(j)$ by setting $P(a_{vj}\;|\;r)$ to a small number ($\epsilon^{2}$ by default).

Finally, paralog-specific genotype probabilities are calculated according to the read-pair location probabilities $p_{r}(i)$ and the read–variant allele observations. Suppose the read-pair *r* has sequence $a_{r}$ aligned to the variant *v*, then we can describe the probability of read-pair *r* according to the paralog-specific genotype $g_{vs}$:


(7) $$P(r\;|\;g_{vs})=\sum_{i=1}^{n}\frac{p_{r}(i)}{c_{si}}\;((1-\epsilon )\cdot \mu \left(a_{r},g_{\mathrm{vsi}}\right)+\epsilon \cdot [c_{si}-\mu \left(a_{r},g_{\mathrm{vsi}}\right)]).$$

Additionally, we can evaluate the probability of each genotype $g_{vs}$ given the set of read pairs $R_{vs}$ that cover the variant *v*:


(8) $$P(g_{vs}\;|\;R_{vs})=\frac{p(g_{vs})\cdot \prod_{r\in R_{vs}}P(r\;|\;g_{vs})}{\sum_{g}p(g)\cdot \prod_{r\in R_{vs}}P(r\;|\;g)}.$$

Paralog-specific genotype probabilities $P(g_{vs}\;|\;R_{vs})$ are later converted into genotype and variant qualities (see Supplementary Methods 2.3). Paralog-specific genotype priors for PSVs are described in Equation (4). For novel non-PSV variants, we define priors $p(g_{vs})$ in the following way:


(9) $$p(g_{vs})=\prod_{i=1}^{n}\{\begin{array}{cc} 1-\xi & \text{if}\;\mu (a_{v}^{*},g_{\mathrm{vsi}})=c_{si},\\ \xi & \text{if}\;\mu (a_{v}^{*},g_{\mathrm{vsi}})\neq c_{si}. \end{array}$$

In other words, each repeat copy genotype $g_{\mathrm{vsi}}$ is penalized by the mutation rate ξ (default: $10^{-3}$) if nonreference allele is present, and where homozygous and heterozygous genotypes are penalized equally.

To reduce the number of false positive variants due to strand-bias in sequencing data (Garrison and Marth 2012), we apply Fisher’s exact test (FET) to the 2×2 table corresponding to the reads counts for the reference and nonreference alleles on the two DNA strands. Variants with a FET *P* value less than .01 are filtered out.

### 2.6 Simulated and real WGS datasets

To benchmark the accuracy of ParascopyVC, we generated two simulated WGS datasets, one with and the other without polymorphic PSVs, which we denote as SIM-Poly and SIM-Fixed, respectively. We did not simulate changes in copy number since variant callers such as DeepVariant (Poplin et al. 2018a)—which we used for benchmarking—only support variant calling in diploid genomes. In case of the SIM-Fixed dataset, we simulated a diploid genome by adding artificial sequence variants every one kilobase on average (80% substitutions, 10% insertions, and 10% deletions; 61.5% heterozygous variants). Rates of different variant types were selected to be similar to the variant rates in the GIAB high-confidence variant calls for the HG002 individual (Zook et al. 2014, 2016, 2019). All simulated variants overlapping PSVs were discarded.

For the SIM-Poly dataset—designed to assess the accuracy of variant calling in the presence of polymorphic PSVs—we simulated WGS reads from a diploid genome with variants that overlapped PSVs. Polymorphic PSVs were simulated according to PSV reference allele frequencies (*f*) in the 503 European ancestry samples from the 1000 Genomes Project (1 kGP) (1000 Genomes Project Consortium et al. 2015), calculated by Parascopy v1.7 (Prodanov and Bansal 2022). Next, we combined the resulting variant set with the SIM-Fixed variant set to obtain $2.75\cdot 10^{6}$ variants. Finally, for both datasets we used ART Illumina (Huang et al. 2012) read simulator tool v2016-06-05 to generate two diploid paired-end datasets for chromosomes 1–22 with 150 bp reads, 30× coverage and mean fragment size = 500 bp. We then used BWA-MEM v0.7.17 (Li and Durbin 2009; Li 2013) to map the simulated reads to the GRCh38 reference genome.

In addition to the simulated WGS datasets, we utilized high-confidence variant call sets for seven human genomes (HG001–HG007) constructed by the GIAB Consortium (Zook et al. 2014, 2016, 2019). The latest version (v4.2.1) of these variant calls leverage long-read sequence datasets to enable accurate variant calls in repetitive regions of the genome that include a large number of LCRs (Wagner et al. 2022). For each individual genome, we analyzed Illumina WGS data that is also made available by the GIAB. The WGS datasets for HG005, HG006, and HG007 genomes had very high coverage (≥100×) and were sub-sampled using samtools v1.14 (Danecek et al. 2021) to obtain ∼30× sequence coverage. We mapped all WGS datasets to the GRCh38 reference genome using BWA-MEM v0.7.17. PacBio HiFi WGS data for HG002 (30-fold coverage, mapped to GRCh38) was also obtained from the GIAB.

### 2.7 Variant calling benchmarking

In order to evaluate variant calling, we utilized a set of 167 LCR loci that we previously compiled for copy number analysis (Prodanov and Bansal 2022). These LCR loci were selected from a genome-wide analysis and each locus in this set overlaps at least one protein-coding gene. Across all repeat copies, the 167 loci span 10.95 Mb of DNA sequence and overlap 380 protein-coding genes. Three variant calling tools—FreeBayes v1.3.5 (Garrison and Marth 2012), GATK HaplotypeCaller v4.2.2 (Poplin et al. 2018b), and DeepVariant v1.4 (Poplin et al. 2018a)—were used for comparison with ParascopyVC. For the HG002 genome, we additionally benchmarked variant calling accuracy on all LCR regions from chromosomes 15, 16, and 17.

We calculated precision and recall using RTG tools v3.12.1 (Cleary et al. 2014, 2015). To compare precision and recall values across different callers, we selected a single variant quality threshold for each caller separately that maximized its average accuracy across seven benchmarking WGS datasets (HG001–HG007). In LCR loci, small precision improvements are achieved at a cost of large decrease in recall; consequently, the best average $F_{1}$ score was obtained at very low-quality thresholds for the three existing variant callers (≤2, corresponding to virtually no filtering). Therefore, we selected optimal variant quality thresholds based on the $F_{0.5}$ score, which favors precision over recall. These criteria produced reasonable quality thresholds of 6, 33, 5, and 21 for GATK, FreeBayes, DeepVariant, and ParascopyVC, respectively. These thresholds were used for comparing precision-recall of different methods across all evaluations.

For ParascopyVC, we calculated aggregate and paralog-specific copy number profiles using Parascopy v1.7 (Prodanov and Bansal 2022) for each dataset (both simulated and real) prior to variant calling. For each dataset, we selected a set of paralog-specific regions that overlap the high-confidence benchmarking regions for the dataset. Additionally, we filtered out LCRs, for which paralog-specific copy number was not estimated by Parascopy (such as LCRs with five or more copies); and excluded LCRs, for which paralog-specific copy number was different than 2. For each sample, benchmarking was limited to the same regions across all variant calling methods.

For running ParascopyVC on the SIM-Fixed dataset, we used PSV allele frequencies equal to 0.9999 to model the fact that PSVs were not polymorphic. For the SIM-Poly dataset, we used PSV allele frequencies obtained from the European ancestry samples in the 1000 Genomes project (Prodanov and Bansal 2022). For the HG001–HG004 and HG005–HG007 datasets, we used PSV allele frequencies obtained from European and East-Asian ancestry samples, respectively. This was consistent with the reported ancestry of these individuals (HG001 is of European ancestry, HG002–HG004 represents an Ashkenazim trio, and HG005–HG007 represent a Han Chinese trio).

To compare the runtime and memory usage for different methods, we called variants for a single individual across 167 duplicated loci. ParascopyVC uses multiple threads to analyze disjoint duplicated loci, so we tabulated run-time using 1 and 16 threads. Similarly, GATK and DeepVariant allow to use multiple threads. FreeBayes uses only one thread, although it can be externally parallelized over independent loci.

## 3 Results

### 3.1 Accuracy of variant calling on simulated data

**Figure 2.**
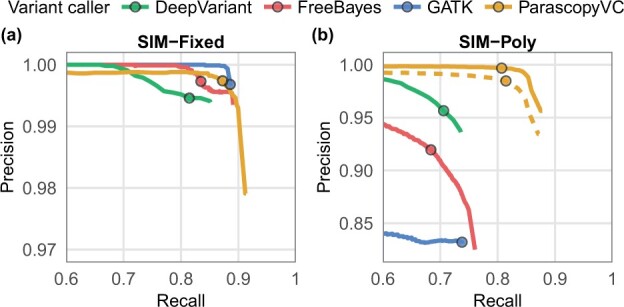
Precision and recall of variant calling for four variant callers (GATK, FreeBayes, DeepVariant, and ParascopyVC) on two simulated WGS datasets. Variant quality thresholds used for comparing precision/recall are marked with circles on each curve. (a) Simulated dataset SIM-Fixed (all PSVs are nonpolymorphic). (b) Simulated dataset SIM-Poly (some PSVs are polymorphic). The solid (dashed) yellow line shows the precision–recall curve for ParascopyVC using PSV allele frequencies from the European (East Asian) population.

For the SIM-Fixed dataset (no polymorphic PSVs), the benchmarking regions covered 9.9 Mb of DNA sequence and included 9254 ground truth variants. On this dataset, FreeBayes, GATK, and ParascopyVC had very high precision (0.997) and similar recall (0.835, 0.886, and 0.872, respectively) (see Fig. 2a). This was not surprising since standard variant calling methods rely on accurately mapped reads and in the absence of polymorphic PSVs and copy number variation, read mapping is reliable. DeepVariant had slightly lower precision (0.995) and recall (0.815) values.

On the SIM-Poly dataset (18 997 baseline variants) that included polymorphic PSVs, the accuracy of state-of-the-art variant callers was drastically lower than for the SIM-Fixed dataset. GATK, FreeBayes, and DeepVariant had low precision (0.832, 0.920, and 0.956, respectively) and recall (0.738, 0.684, and 0.706) (see Fig. 2b). ParascopyVC significantly outperformed all methods and achieved high recall (0.807) and very high precision (0.997).

Since ParascopyVC uses population reference allele frequencies of PSVs for variant calling, the choice of population can potentially impact the accuracy of variant calling. To assess this, we ran ParascopyVC using PSV allele frequencies derived from the East Asian population in the 1000 Genomes Project. ParascopyVC’s accuracy was slightly lower (dashed yellow line in Fig. 2b) than the accuracy using European allele frequencies but was significantly better than all other methods.

### 3.2 Accuracy of variant calling on the HG002 genome

The GIAB has compiled high-confidence variant call sets for seven human individuals by careful aggregation of variant call sets derived from numerous variant calling tools and multiple sequencing technologies (Zook et al. 2014, 2016, 2019). The HG002 genome is the most studied of the seven GIAB datasets and therefore, we first benchmarked our method on this genome. The overlap between the GIAB high-confidence benchmarking regions for the HG002 genome and the 167 LCR loci covered 7.35 Mb of DNA sequence. After excluded regions where Parascopy estimated nonreference paralog-specific copy number (0.39 Mb) or was unable to estimate paralog-specific copy number (0.70 Mb), the benchmarking regions covered 6.26 Mb of DNA sequence and contained 7985 ground truth variants.

**Figure 3.**
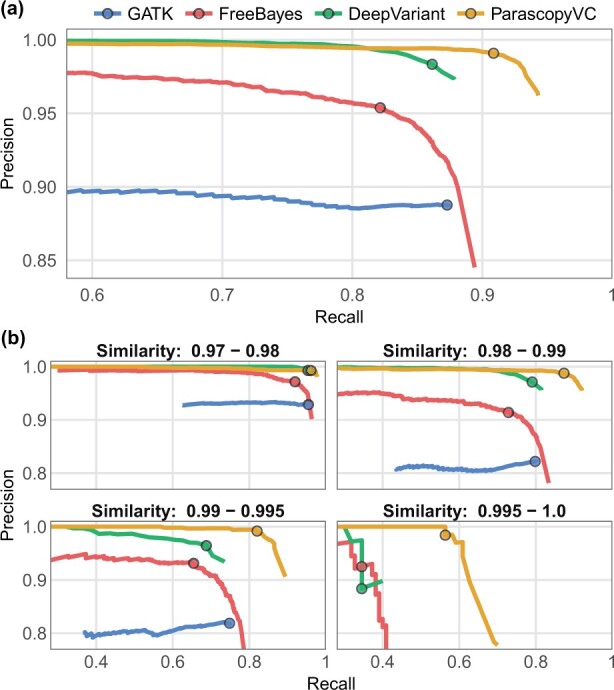
Precision and recall of variant calling using four callers (GATK, FreeBayes, DeepVariant, ParascopyVC) on the HG002 benchmark dataset. For each caller, circles denote the quality thresholds that maximize the average accuracy across seven human genomes. (a) Precision–recall values across all LCR regions. (b) Precision–recall values across LCR regions split into four groups based on sequence similarity (0.97–0.98, 0.98–0.99, 0.99–0.995, and 0.995–1.0).

Comparison of the variant calling accuracy for the four methods (Fig. 3a and Supplementary Table S1) showed that Freebayes and GATK ($F_{1}$ scores of 0.883 and 0.880 respectively) had much lower precision and recall values than DeepVariant and ParascopyVC. ParascopyVC correctly called 7255 variants (recall = 0.909) and incorrectly called only 65 variants (precision = 0.991), resulting in a very high $F_{1}$ score of 0.948. In comparison, DeepVariant could achieve high precision (0.983) but had lower recall (0.861) than ParascopyVC, achieving $F_{1}$ score of 0.918. For comparison, we also evaluated the accuracy of DeepVariant calls on 30× long-read HiFi WGS data for the HG002 genome. The HiFi calls had very high precision (0.9985, 12 false positives) and recall (0.991, 73 false negatives).

Comparison of the accuracy for SNVs and short indels separately showed that ParascopyVC achieved the best precision (0.995) and best recall (0.914) among all methods for SNVs (Table 1). ParascopyVC’s SNV $F_{1}$ score was 0.953 while the next best $F_{1}$ score (0.919) was achieved by DeepVariant. For indels, DeepVariant had higher precision (0.978) compared to ParascopyVC (0.972) while both methods had the same recall.

**Table 1.** Variant calling accuracy for single-nucleotide variants (SNVs) and insertions or deletions (indels) on the HG002 benchmarking WGS dataset.

Variants	Method	FP	FN	Precision	Recall	$F_{1}$
SNPs	GATK	821	920	0.887	0.875	0.881
	FreeBayes	276	1263	0.954	0.828	0.887
	DeepVariant	104	1017	0.984	0.862	0.919
	ParascopyVC	**31**	**634**	**0.995**	**0.914**	**0.953**
Indels	GATK	62	95	0.895	0.848	0.871
	FreeBayes	33	147	0.950	0.765	0.848
	DeepVariant	**12**	**90**	**0.978**	**0.856**	**0.913**
	ParascopyVC	34	**90**	0.972	**0.856**	0.911

The table shows the number of false positive (FP) and negative (FN) variant calls, precision, recall, and $F_{1}$ scores for 7361 baseline SNPs and 624 baseline indels. Best value in each subtable is printed in bold.

Next, we evaluated the accuracy of variant calling in LCR regions stratified by sequence similarity. For this, we split the LCR regions into four groups (sequence similarity in the range 0.97–0.98, 0.98–0.99, 0.99–0.995, and 0.995–1.0). The comparison of precision-recall for the different methods showed that ParascopyVC’s accuracy, was greater for LCR regions with higher sequence similarity relative to other methods (Fig. 3b). Compared to the other three variant callers, ParascopyVC maintained high precision across LCR regions with different sequence similarity. The HG002 high-confidence variant calls included 1016 variants in LCR regions with ≥99% sequence similarity. In these regions, ParascopyVC had a very high precision (0.991) and high recall (0.792). DeepVariant’s recall (0.651) and precision (0.959) in these regions were lower compared to its overall recall (0.861) and precision (0.983). The high recall for ParascopyVC in LCR regions with very high sequence similarity is likely because it utilizes all reads (even with a mapping quality of zero) for variant discovery and genotyping.

To assess the accuracy of ParascopyVC in noncoding LCRs, we performed variant calling in all LCR regions from chromosomes 15, 16, and 17. The benchmarking regions covered 5.85 Mb of DNA sequence and included 6724 high-confidence variants. The relative performance of the different variant callers was similar to that observed in the 167 coding LCRs. ParascopyVC had the best overall precision (0.982) and recall (0.864), DeepVariant achieved similar precision (0.975) but lower recall (0.805) while GATK and Freebayes had much lower accuracy than DeepVariant and ParascopyVC (see Supplementary Table S2).

### 3.3 Benchmarking variant calling on additional human genomes

**Figure 4.**
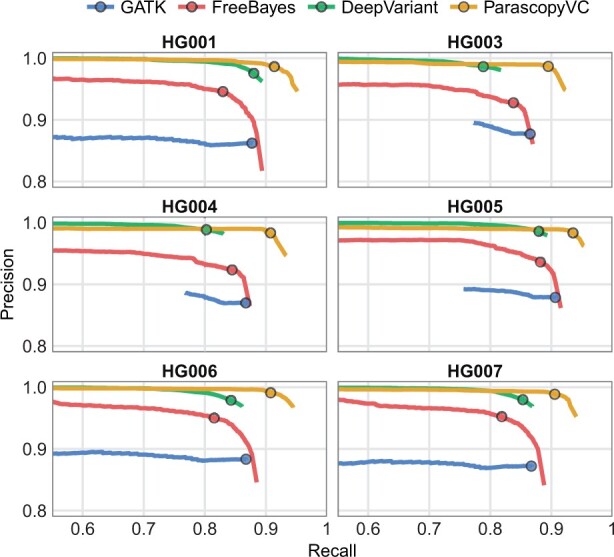
Precision and recall of variant calling using four callers (GATK, FreeBayes, DeepVariant, ParascopyVC) in LCR regions across six human genomes. For each caller, circles denote the quality thresholds that maximize the average accuracy across seven genomes (including HG002).

We benchmarked ParascopyVC on the six additional human genomes for which the GIAB consortium has generated high-confidence variant calls. Benchmarking regions across all seven GIAB variant call sets (HG001–HG007) covered between 6.26 Mb and 6.46 Mb of LCR regions and contained between 7.8 and 9.5 thousand variants (Supplementary Table S1). The precision-recall curves for the six genomes (Fig. 4) were very similar to that for the HG002 genome and showed that ParascopyVC had consistently higher precision and recall than both GATK and Freebayes. The average precision for ParascopyVC across the seven genomes was 0.987 compared to 0.876, 0.941, and 0.983 for GATK, Freebayes, and DeepVariant, respectively (Supplementary Table S1). Although the precision for DeepVariant was comparable to that for ParascopyVC, the average recall for DeepVariant (0.844) was much lower than that for ParascopyVC (0.911).

### 3.4 Run time and memory usage

On a single individual (HG002) and 167 duplicated loci, FreeBayes called variants in 04:41 (mm: ss) and used approximately 0.5 Gb memory. GATK HaplotypeCaller consumed 10 Gb memory and called variants in 10:21 (1 thread) or 08:57 (16 threads). DeepVariant had similar performance: 3 Gb memory usage and a run-time of 13:09 (1 thread) or 3:25 (16 threads). ParascopyVC required 7 Gb memory and called variants in 80:28 (1 thread) or 16:36 (16 threads). In contrast to existing variant calling tools which run diploid variant calling for human genomes, ParascopyVC performs polyploid variant calling using FreeBayes. Polyploid variant calling and genotyping requires more calculations than diploid variant calling since the number of possible genotypes for a variant site is combinatorial in the ploidy and number of alleles. Consequently, ParascopyVC requires more time compared to other methods.

## 4 Discussion

In this article, we have described a new computational method specifically designed for variant calling in LCRs using WGS data. We have demonstrated—using both simulated and real WGS datasets—that this approach significantly improves accuracy of variant calling in LCRs in human genomes. Compared to existing variant callers that call variants in each copy of LCRs separately, ParascopyVC jointly analyzes reads mapped to all repeat copies to first identify candidate variants. Although several methods have previously utilized a similar strategy of aggregating read information for variant calling in LCRs (Kerzendorfer et al. 2015; Ebbert et al. 2019), ParascopyVC implements an end-to-end variant calling approach that starts from aligned reads (BAM/CRAM files) as input and output variant calls (VCF files). A key feature of ParascopyVC is that it does not assume correctness of the reference genome at PSVs for variant calling. Instead, it leverages population-based reference allele frequencies and read-level information at PSVs to identify those PSVs that can be used to distinguish repeat copies.

Benchmarking of multiple variant callers in LCR regions showed that DeepVariant had higher precision than both GATK and FreeBayes. DeepVariant is a neural network based variant caller that has been trained using GIAB benchmark calls for human genomes. As per the DeepVariant github FAQ, DeepVariant has potentially learned to filter out false positives in LCRs based on signatures such as excessive read depth, allele bias, or clustering of variants. Nevertheless, ParascopyVC obtains significantly higher recall than DeepVariant while achieving similar precision indicating that direct modeling of LCRs enables high precision and recall.

ParascopyVC has been developed to complement existing variant calling tools in LCR regions and not replace them. Indeed, it leverages an existing variant caller, FreeBayes, for variant discovery. Currently, it uses a simple constant error-rate based model for calculating genotype likelihoods for indels. This reduces the indel calling precision, particularly in low-complexity sequences with high indel error rate. The indel calling precision can be improved by leveraging error models or annotations from existing variant callers.

ParascopyVC leverages population reference allele frequencies for PSVs for variant calling. As a result, it does not call variants in regions for which these frequencies are not available. In such regions, variant calling using existing state-of-the-art tools is likely to have low accuracy since a large fraction of PSVs are polymorphic. Our previous analysis of 167 LCR loci (Prodanov and Bansal 2022) using 1000 Genomes WGS data has shown that PSV frequencies cannot be estimated for 10–15% of loci. In the future, as long read sequencing data and high-quality genome assemblies become available for a large number of human genomes (Wang et al. 2022), these can potentially be used to obtain PSV reference allele frequencies for such loci and improve the accuracy of variant calling.

ParascopyVC uses estimates of paralog-specific copy number as input and can call variants in the presence of copy number variants. However, we did not evaluate its accuracy in the presence of copy number changes in this article. Benchmarking variant calling in the presence of copy number variants is challenging with short reads since it requires accurate estimates of copy number in addition to accurate variant calls. We plan to explore this in future work.

## Supplementary Material

btad268_Supplementary_Data

## Data Availability

The analyses presented in this article are based on the high-coverage whole genome sequencing data of 1000 Genomes Project samples, available via ENA Study PRJEB31736 and PRJEB36890.
