# Supplementary material for: A multilocus approach for accurate variant calling in low-copy repeats using whole-genome sequencing
Source: Bioinformatics. 2023 Jun 30;39(Suppl 1):i279–87. doi: 10.1093/bioinformatics/btad268 (PMC10311303; doi:10.1093/bioinformatics/btad268)
Supplement: btad268_Supplementary_Data [file btad268_supplementary_data.pdf]

A multi-locus approach for accurate variant calling in low-copy repeats using whole-genome sequencing

Supplementary Materials

Timofey Prodanov [1,2,3,*] and Vikas Bansal [4,*]

[1] Bioinformatics and Systems Biology Graduate Program, University of California San Diego, La Jolla, CA 92093, USA. [2] Institute for Medical Biometry and Bioinformatics, Medical Faculty, Heinrich Heine University, Düsseldorf 40225, Germany. [3] Center for Digital Medicine, Heinrich Heine University, Düsseldorf 40225, Germany. [4] School of Medicine, University of California San Diego, La Jolla, CA 92093, USA.

[*] **Contact:** timofey.prodanov@hhu.de and vibansal@ucsd.edu

1 Supplementary Tables

Supplementary Table 1: **Comparison of the precision, recall and F_1 scores for variant calling on seven WGS datasets from the GIAB.** The first two columns show the WGS sample and the number of benchmarking variants. Best precision, recall and F_1 score for each sample are printed in bold.

Genome	Variants	GATK			FreeBayes			DeepVariant			ParascopyVC		
		prec.	recall	F_1	prec.	recall	F_1	prec.	recall	F_1	prec.	recall	F_1
HG001	7 786	0.862	0.877	0.870	0.946	0.830	0.884	0.976	0.880	0.925	**0.987**	**0.914**	**0.949**
HG002	7 985	0.888	0.873	0.880	0.954	0.822	0.883	0.983	0.861	0.918	**0.991**	**0.909**	**0.948**
HG003	7 967	0.877	0.865	0.871	0.928	0.838	0.881	0.986	0.788	0.876	**0.987**	**0.895**	**0.939**
HG004	8 732	0.870	0.867	0.869	0.924	0.845	0.882	**0.989**	0.803	0.886	0.984	**0.908**	**0.944**
HG005	9 275	0.879	0.906	0.892	0.936	0.882	0.909	**0.986**	0.880	0.930	0.983	**0.935**	**0.959**
HG006	9 002	0.883	0.867	0.875	0.950	0.815	0.878	0.979	0.843	0.906	**0.991**	**0.908**	**0.948**
HG007	9 480	0.872	0.867	0.870	0.953	0.819	0.881	0.980	0.853	0.912	**0.989**	**0.906**	**0.945**
Average		0.876	0.875	0.875	0.941	0.836	0.885	0.983	0.844	0.908	**0.987**	**0.911**	**0.947**

Supplementary Table 2: **Variant calling accuracy at all low-copy repeats on chromosomes 15, 16 and 17 on the HG002 benchmarking WGS dataset.** The table shows the number of false positive (FP), and negative (FN) variant calls, precision, recall and F_1 scores for 6,724 baseline variants. Benchmarking regions cover 5.85 Mb of the genome. Best value in each column is printed in bold.

Method	FP	FN	Precision	Recall	F_1
GATK	929	1197	0.856	0.822	0.839
FreeBayes	332	1588	0.938	0.764	0.842
DeepVariant	140	1314	0.975	0.805	0.882
ParascopyVC	**104**	**918**	**0.982**	**0.864**	**0.919**

2 Supplementary Methods

2.1 Unknown paralog-specific copy number

It is possible that sample paralog-specific copy number is not fully known: for example in a three-copy duplication sample aggregate copy number was identified as $\hat{c}_s = 6$ and paralog-specific copy number as $c_s = (2, ?, ?)$. In such cases we virtually combine repeat copies with unknown paralog-specific copy numbers into a new, *extended* repeat copy.

It is possible that several paralog-specific and several aggregate genotypes are reference-compatible if a PSV v has different reference alleles within a single extended repeat copy. Consequently, we discard informative PSVs, if one of the alleles appears in the reference sequences of all extended repeat copies.

2.2 Identifying possible locations for sequencing reads in LCRs

In order to identify possible sequencing read locations, we utilize the overlaps between reads, sequence variants, and paralogous sequence variants (PSVs). For each variant ParascopyVC stores information about its positions across all repeats copies. For a given read we evaluate the variants covered by the read, combine all variant positions across all repeat copies, and sort them by the genomic coordinate. Next, we select such clusters of variant coordinates that the distance between the first and last coordinate does not exceed the read length by more than 10 bp. Additionally, we combine two clusters of variant coordinates into one, if the distance between them is smaller than 50 bp, but assume that such cluster cannot represent a correct read location. Forbidden read locations receive 10^{-20} location probability penalty.

In certain cases, we can either discard one of the possible locations, or be certain that the original location is correct. Consider a read with an *original* alignment to one of the repeat copies, that was remapped to another repeat copy to get a *pooled* alignment. We say that an alignment has unique tail if it contains at least 15 bp that do not overlap any entry in the homology table. If the original alignment has high mapping quality (≥ 50), has a unique tail, and has the same or fewer clipped basepairs than the pooled alignment (or if the original alignment matches the pooled alignment and has no clipping at all) — we say that the original alignment location is certainly correct. Finally, if the original alignment is much better than the pooled alignment (aligned length is at least 15 bp more) — we say that the pooled alignment is certainly *incorrect*. We cannot easily confirm that the original location is correct, as it is possible that there exists

a third repeat copy that the read can map to, and checking for such cases would significantly hamper the execution time.

2.3 Output files and quality scores

ParascopyVC generates two output variant call format (VCF) files: aggregate and paralog-specific. In an aggregate VCF file, reference allele of a sequence variant or PSV is the reference sequence of the variant in the first repeat copy of the duplication. In case of PSVs, the reference alleles from all other repeat copies are stored as alternative alleles. For each sample, ParascopyVC provides the most probable aggregate genotype $\hat{g}_{vs}$ and its Phred quality score, calculated as $Q(\hat{g}_{vs}) = -10 \cdot \log_{10}\left(1 - P(\hat{g}_{vs})\right)$.

In a paralog-specifc VCF file, ParascopyVC outputs variants once for each repeat copy. For each sample and each repeat copy i, ParascopyVC finds the most probable marginal paralog-specific genotype $g_{vs}^{(i)}$, where $P(g_{vs}^{(i)}) = \sum_{g' \text{ s.t. } g'_i = g_{vs}^{(i)}} P(g')$. Phred quality scores for the marginal paralog-specific genotypes are calculated in a similar manner to the aggregate genotype qualities: $Q(g_{vs}^{(i)}) = -10 \cdot \log_{10}\left(1 - P(g_{vs}^{(i)})\right)$.

Each variant in a variant call VCF is characterized by its variant quality, which is a different metric compared to the genotype qualities. Traditionally, variant quality encodes a probability that the variant genotype contains a non-reference allele for at least one of the samples. In an aggregate output VCF file, ParascopyVC sets PSV qualities to a high constant value based on the following two cases: (i) if an aggregate PSV genotype is *reference-compatible*, then it contains a non-reference allele compared to the first repeat copy; (ii) if an aggregate PSV genotype is not reference-compatible, then it contains a non-reference allele in one of the marginal paralog-specific genotypes. For all sequence variants that do not overlap PSVs, ParascopyVC uses underlying FreeBayes quality scores as the variant qualities in the aggregate VCF file.

In the paralog-specific output VCF file, ParascopyVC uses the same formula to calculate variant qualities for both sequence variants and PSVs: $Q^{(i)}(v) = -10 \sum_{s \in S} \log_{10} P(g_{vsi}^{*})$, where g_{vsi}^{*} is the reference paralog-specific genotype on the repeat copy i (consisting entirely of the reference allele a_{vi}^{*}). In other words, ParascopyVC calculates the Phred quality score of the probability that all samples exhibit the reference paralog-specific genotype on the repeat copy i.
